# Congenital Viral Infections of the Brain: Lessons Learned from Lymphocytic Choriomeningitis Virus in the Neonatal Rat

**DOI:** 10.1371/journal.ppat.0030149

**Published:** 2007-11-30

**Authors:** Daniel J Bonthius, Stanley Perlman

**Affiliations:** University of British Columbia, Canada

## Abstract

The fetal brain is highly vulnerable to teratogens, including many infectious agents. As a consequence of prenatal infection, many children suffer severe and permanent brain injury and dysfunction. Because most animal models of congenital brain infection do not strongly mirror human disease, the models are highly limited in their abilities to shed light on the pathogenesis of these diseases. The animal model for congenital lymphocytic choriomeningitis virus (LCMV) infection, however, does not suffer from this limitation. LCMV is a well-known human pathogen. When the infection occurs during pregnancy, the virus can infect the fetus, and the developing brain is particularly vulnerable. Children with congenital LCMV infection often have substantial neurological deficits. The neonatal rat inoculated with LCMV is a superb model system of human congenital LCMV infection. Virtually all of the neuropathologic changes observed in humans congenitally infected with LCMV, including microencephaly, encephalomalacia, chorioretinitis, porencephalic cysts, neuronal migration disturbances, periventricular infection, and cerebellar hypoplasia, are reproduced in the rat model. Within the developing rat brain, LCMV selectively targets mitotically active neuronal precursors. Thus, the targets of infection and sites of pathology depend on host age at the time of infection. The rat model has further shown that the pathogenic changes induced by LCMV infection are both virus-mediated and immune-mediated. Furthermore, different brain regions simultaneously infected with LCMV can undergo widely different pathologic changes, reflecting different brain region–virus–immune system interactions. Because the neonatal rat inoculated with LCMV so faithfully reproduces the diverse neuropathology observed in the human counterpart, the rat model system is a highly valuable tool for the study of congenital LCMV infection and of all prenatal brain infections In addition, because LCMV induces delayed-onset neuronal loss after the virus has been cleared, the neonatal rat infected with LCMV may be an excellent model system to study neurodegenerative or psychiatric diseases whose etiologies are hypothesized to be virus-induced, such as autism, schizophrenia, and temporal lobe epilepsy.

## Introduction

Multiple viruses, bacteria, and parasites can infect the developing human fetus, resulting in a range of outcomes that include fetal demise, developmental anomalies, and disease of the newborn [1]. Disease may also be clinically silent at birth and not become evident until after the first few months or years of life. Factors important in determining outcome include pathogen identity, gestational timing of infection, pathogen load, tissue tropism, inflammatory response, and immune status of the mother and fetus.

For example, human infection with the rubella virus during the first 11 weeks of gestation results in teratogenic changes in most fetuses that survive the acute infection, with abnormalities commonly detected in the heart, eye, and central nervous system [2]. At later times in gestation (11–16 weeks), infection is less likely to result in congenital anomalies, but may still result in hearing loss, mental retardation, and growth deficits [3]. Sequelae from congenital rubella may become manifest at even later times of postnatal life, with insulin-dependent diabetes apparent in up to 20% of infected humans by adulthood [4]. Although congenital infections caused by rubella virus have been greatly decreased as a consequence of vaccination, effective vaccines are not available for infections caused by other pathogens, such as cytomegalovirus, which are also important causes of congenital infection.

Perinatal infection by less common or emerging pathogens may become increasingly prevalent. One such emerging viral pathogen is lymphocytic choriomeningitis virus (LCMV), an arenavirus that has been increasingly recognized as a teratogen in recent years [5–11].

LCMV was initially isolated by Armstrong and Lillie in 1933 [12] from the cerebrospinal fluid of a woman who was thought to have St. Louis encephalitis. This patient had presented with general malaise, but her condition worsened, and she died. The virus isolated from her cerebrospinal fluid was passaged five times through monkeys and, with each passage, produced a disease resembling St. Louis encephalitis. On the sixth passage, the virus was inoculated into a monkey that was immune to St. Louis encephalitis. However, the virus still produced the disease, indicating that the virus was not St. Louis encephalitis virus. This new infectious agent was named “lymphocytic choriomeningitis virus” for the pathologic changes that it induced in the choroid plexus and meninges of infected mice and monkeys [12].

The virus was subsequently isolated from cerebrospinal fluid of multiple patients with aseptic meningitis. Thus, it was established that LCMV was an important etiologic agent of aseptic meningitis in humans. Subsequent clinical and etiologic studies identified LCMV as one of the most frequent infectious causes of aseptic meningitis in humans [13].

The first recognized case of congenital infection with LCMV was reported in England in 1955 [14]. In the decades that followed, multiple cases of congenital LCMV infection were reported throughout Europe [15,16]. Although LCMV has been recognized as an important cause of aseptic meningitis in the United States for decades, the first cases of congenital LCMV infection were not reported in the United States until 1993 [17,18].

Like all arenaviruses, LCMV utilizes rodents as its principal reservoir [19–21]. Mus musculus, the common house mouse, is both the natural host and reservoir for the virus, which is transferred vertically from one generation to the next within the mouse population by intrauterine infection. Although heavily infected with LCMV, mice that acquire the virus prenatally often remain asymptomatic because the virus is not cytolytic and because congenital infection provides the mice with immunological tolerance for the virus [22,23]. Throughout their lives, mice prenatally infected with LCMV shed the virus in large quantities in nasal secretions, saliva, milk, semen, urine, and feces.

Postnatal humans acquire LCMV by inhalation of aerosolized virus or by direct contact with fomites contaminated with infectious virus. LCMV infection during postnatal life (childhood or adulthood) typically consists of a brief febrile illness from which the patient fully recovers. Symptoms include headache, fever, myalgia, photophobia, and vomiting. In as many as one-third of postnatal infections, the disease is asymptomatic.

Human-to-human horizontal infection has not been documented, except for the unusual circumstances in which the virus was acquired through transplantation of infected tissues [24]. In contrast, human-to-human vertical transmission does occur and is the basis for congenital LCMV infection.

Because LCMV is prevalent in the environment and has a great geographic range, the virus infects large numbers of humans. An epidemiologic study has demonstrated that 9% of house mice in urban Baltimore are infected with LCMV, and that substantial clustering occurs where the prevalence is higher [25]. Serological studies have demonstrated that 5.1% of healthy black women in Birmingham and 4.7% of adults in Baltimore possess antibodies to LCMV, indicating prior exposure and infection [25,26].

The prevalence and incidence of congenital LCMV infection are unknown. While case reports of infection during pregnancy demonstrate that LCMV can induce severe defects in brain structure and function [6–8,11,14,15], it is not known whether the profoundly affected infants described in the case reports represent the typical outcome of gestational LCMV infection, or whether they represent only the most severely affected cases. Prospective clinical or epidemiological studies of congenital LCMV infection have not been conducted. The fact that LCMV is not one of the infectious agents for which infants with a suspected congenital infection are routinely evaluated further limits information regarding the incidence and spectrum of LCMV-induced teratogenicity. Therefore, congenital LCMV infection might produce a spectrum of pathologic effects ranging from minimal to severe [11]. The high prevalence of infected mice and of sero-positive postnatal humans suggest that congenital LCMV infection is an underdiagnosed disease and that the virus is responsible for more cases of congenital neurologic and vision dysfunction than has previously been recognized [6,7,27,28].

Transplacental infection of the fetus is the basis for most cases of congenital LCMV infection, presumably during maternal viremia [5]. In some cases, the fetus may acquire LCMV during the intrapartum period [14]. Within the human fetus, the brain is the principal target of LCMV infection and the most important site of pathology [8,11]. Mitotically active neuronal precursors are particularly vulnerable to LCMV infection and an important site of LCMV replication [5,29,30]. Microencephaly, periventricular calcifications, gyral dysplasia, cerebellar hypoplasia, and focal cerebral destruction are common pathologic effects of congenital LCMV infection [11]. These pathologic changes reflect both the viral tropism for replicating neuroblasts and disrupted brain development induced by loss or dysfunction of immature or replicating neurons [5,8,30,31]. The mechanism by which LCMV damages the fetal human brain is unknown. LCMV is not a cytolytic virus in most cell types, including neurons. Thus, unlike herpes and several other pathogens that directly damage the brain by killing host brain cells [1], LCMV neuroteratogenicity must have some other underlying pathogenesis [30].

**Figure 1:**
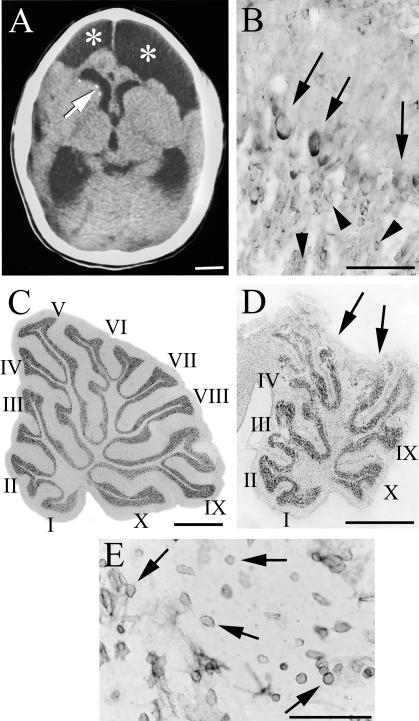
LCMV Infection Induces Focal Destructive Lesions within the Developing Brains of Humans and Rats (A) Head CT scan from a 4-mo-old child with congenital LCMV infection. The scan reveals bilateral asymmetric regions of encephalomalacia (asterisks), strongly suggestive of a focal destructive process. Note also the periventricular calcifications (arrow), characterisitic of a prenatal viral infection. (B) Section (50-μm-thick) through the cerebellar cortex of a neonatal rat infected with LCMV. The section has been immunohistochemically stained for LCMV antigens. The virus infects both Purkinje cells (arrows) and granule cells (arrowheads). (C) Nissl-stained section (2-μm-thick) through the cerebellar vermis of an uninfected (control) 30-d-old rat. The ten lobules of the cerebellar vermis (I–X) are labelled according to the system of Larsell [84]. (D) Section (2-μm-thick) through the cerebellar vermis of a 30-d-old rat infected 3 wk earlier with LCMV. The dorsal cerebellum has undergone a destructive process (arrows). Most of lobules V, VI, VII, and VIII have been obliterated, while lobules I, II, III, and X have been relatively spared. (E) Section (50-μm-thick) through the cerebellar cortex of a 14-d-old rat infected 10 d earlier with LCMV. The animal was sacrificed at the time that acute destruction of the cerebellum was occurring. The section has been immunohistochemically stained for CD8+ antigen, which labels a subset of lymphocytes. Note the dense infiltration of CD8+ lymphocytes (arrows). Magnification bars represent 1 cm in (A), 100 um in (B), 500 um in (C), 500 um in (D), and 100 um in (E).

**Figure 2:**
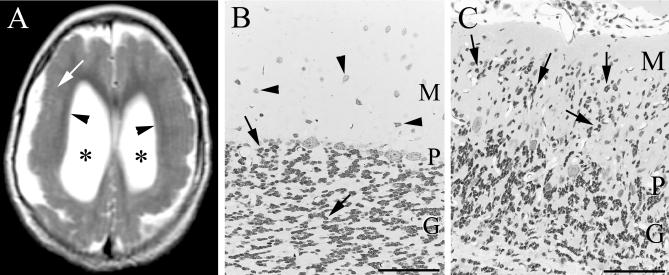
LCMV Infection Disrupts Neuronal Migration in the Developing Brain of Humans and Rats (A) MRI scan of a 3-y-old child with congenital LCMV infection. The MRI scan demonstrates microencephaly and a deficit of white matter (arrowheads) with a compensatory enlargement of the lateral ventricles (asterisks). There is also a diminished number of cortical sulci and an abnormally smooth cortical surface (white arrow). This is strongly suggestive of pachygyria, a developmental defect due to abnormal neuronal migration. (B and C) are 2-μm-thick sections through the cerebellar cortex of uninfected control (B) and LCMV-infected (C) rats. (B) Normal cerebellar cortex from a control (uninfected) adult rat demonstrating the trilaminar cytoarchitecture of the cortex, which consists of the molecular layer (M), Purkinje cell layer (P), and granule cell layer (G). Within the molecular layer, a few stellate cells and basket cells (arrowheads) are normally present. In contrast, granule cells (arrows) have migrated through the molecular layer to the granule cell layer. Granule cells no longer reside in the molecular layer in the normal cerebellum. (C) Cerebellar cortex from an adult rat infected during early postnatal life with LCMV. Many granule cells (arrows) remain abnormally placed within the molecular layer. As a result of LCMV infection, these neurons have failed to migrate properly to their normal location within the granule cell layer and remain permanently ectopic within the molecular layer. Magnification bars represent 100 um in (B and C).

**Figure 3:**
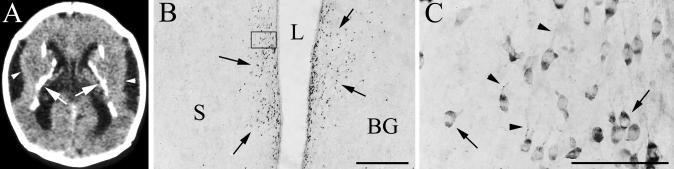
Congenital LCMV Infection of Humans Induces Periventricular Calcifications The neonatal rat model demonstrates that periventricular neurons are selectively vulnerable to infection with LCMV. (A) Head CT scan from an infant with congenital LCMV infection. The scan reveals microencephaly and prominent periventricular calcifications (arrows). In addition, this scan reveals an abnormal cortical gyral pattern (arrowheads), suggestive of disturbed cortical neuronal migration. (B) Horizontal section (50-μm-thick) through a 49-d-old rat brain immunohistochemically stained for LCMV. The rat was inoculated as a neonate with LCMV. Infection is localized to the periventricular region (arrows). L = lateral ventricle, S = septum, BG = basal ganglia. (C) Higher magnification of the boxed area in (B) shows that the infected cells are neuronal in morphology. Viral antigen is present in neuronal cell bodies (arrows) and neurites (arrowheads). Magnification bars represent 500 um in (B) and 100 um in (C).

Progress toward understanding the pathogenesis of most perinatal infections is limited by the absence of animal models that mirror human disease. However, this is not the case for congenital LCMV infection. Neonatal rats infected with LCMV develop virtually all of the neuropathological abnormalities observed in infected humans [29,30], including, but not limited to encephalomalacia (Figure 1), disrupted neuronal migration (Figure 2), and periventricular infection (Figure 3). Most strikingly, the rat model of LCMV illustrates the complex interactions among timing of infection relative to animal age, cellular tropism, and the host immune response in determining acute disease and ultimate outcome [30].

In the rat model, rat pups receive intracerebral injections of LCMV during the neonatal period [30–32]. Postnatal rat pups model human congenital LCMV infection because the rat brain is immature at the time of birth, relative to the human brain [33]. Thus, in terms of brain development, the first two postnatal weeks in the rat mimic the second half of human gestation [34].

The neonatal rat model of congenital LCMV infection was pioneered by Monjan and coworkers during the 1970s and early 1980s. They discovered that LCMV induces a distinct pattern of infection in which certain neuronal populations are infected, while others are spared [31,32]. They further found that the virus can induce retinopathy [35], cerebellar destruction [32], disrupted neurotransmission [36], and altered behavior [37]. In addition, they found that much of the pathology induced by LCMV is immune-mediated [38]. This finding of an important role for the immune system in LCMV infection of the neonatal rat complemented the many landmark studies by others over the past 70 years, in which immunologists and virologists have utilized LCMV to gain a deeper understanding of immunology and immunopathology [39–41]. Recently, interest in the neonatal rat model of LCMV infection has been re-ignited, as the importance of human congenital LCMV infection and the value of the rat model system for studying that infection have become clear.

## Critical Role of Host Age in LCMV-Induced Disease

**Figure 4:**
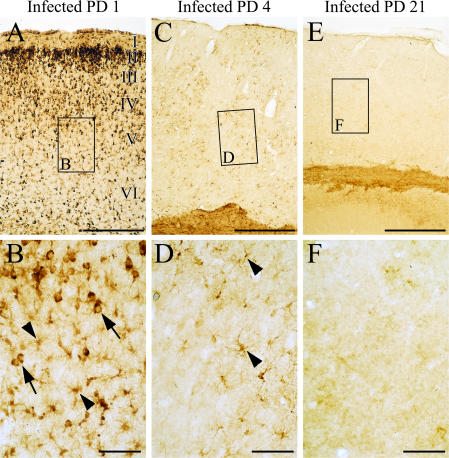
The Cellular Targets of LCMV within the Cerebral Cortex Depend on Host Age Shown are 50-μm-thick sections through the cerebral cortex of rats infected on PD 1 (A), PD 4 (C), or PD 21 (E). In each case, the animals were sacrificed 25 d post-inoculation, and the brain sections were immunohistochemically stained for LCMV antigens. Inoculation on PD 1 leads to infection of cerebral cortical neurons (arrows) and astrocytes (arrowheads). Note that a greater proportion of neurons are infected in superficial cortical layers than in deep cortical layers. Inoculation on PD 4 leads to infection of astrocytes alone (arrowheads). Whereas countless neurons were infectable 3 d earlier, virtually no neurons are infectable by PD4. Inoculation on PD 21 leads to infection of neither neurons nor astrocytes.

In the rat model, host age profoundly affects the outcome of LCMV infection [30]. The cellular targets of infection, maximal viral titers, and the nature and the severity of neuropathology all depend strongly on host age at the time of infection. Figure 4 illustrates the importance of host age in determining the cellular targets of infection. Inoculation of the rat pup on postnatal day (PD) 1 leads to a widespread infection of the cerebral cortex, in which both astrocytes and neurons are infected. However, if the infection occurs just 3 d later, on PD4, only astrocytes are infected, and neurons are completely spared. By PD21, no cells of the cerebral cortex—neither astrocytes nor neurons—are infectable with LCMV. Thus, over the course of a short period of developmental time, the cellular targets of infection within the cerebral cortex change from a combination of astrocytes and neurons, to astrocytes alone, to no infectable cells [30].

This progressive restriction in the cellular targets of infection is reflected by a decline in the maximal viral titers. When the infection occurs on PD1, maximal viral titer exceeds 10^8^ plaque-forming units per gram of tissue. By PD4, maximal viral titer falls 100-fold to 10^6^, and by PD21, no live virus is detectable in the cerebral cortex (though the virus remains detectable in some other brain regions, including the olfactory bulb and ventricular ependyma).

The neuropathology induced by LCMV likewise depends strongly on host age [30]. Specific neuropathologic changes are reliably produced by infection at certain ages and reliably absent at others. For example, LCMV induces cerebellar hypoplasia, in which the cerebellum is small but has a normal cytoarchitecture, only if the infection occurs on PD1. In contrast, if the infection occurs on PD4 or PD6, then LCMV induces a neuronal migration disturbance, in which the cerebellar granule cells fail to migrate properly in the ventral lobules, and encephalomalacia, in which the dorsal lobules undergo a destructive process and are obliterated. In further contrast, if the infection occurs on PD21, then LCMV induces no cerebellar pathology at all (though it continues to induce olfactory bulb destruction and hydrocephalus, because of the continued infectability of the olfactory bulb and ventricular ependyma).

Children congenitally infected with LCMV can have a diverse set of neuropathologic changes that vary from case to case [11]. Virtually all of these neuropathologic changes observed among children with congenital LCMV infection can be recapitulated in the rat model by infecting the host at different ages (Table 1). This finding strongly suggests that the diversity in pathology and outcome among children with congenital LCMV infection is due, at least in part, to differences in gestational age at the time of infection.

**Table 1:**
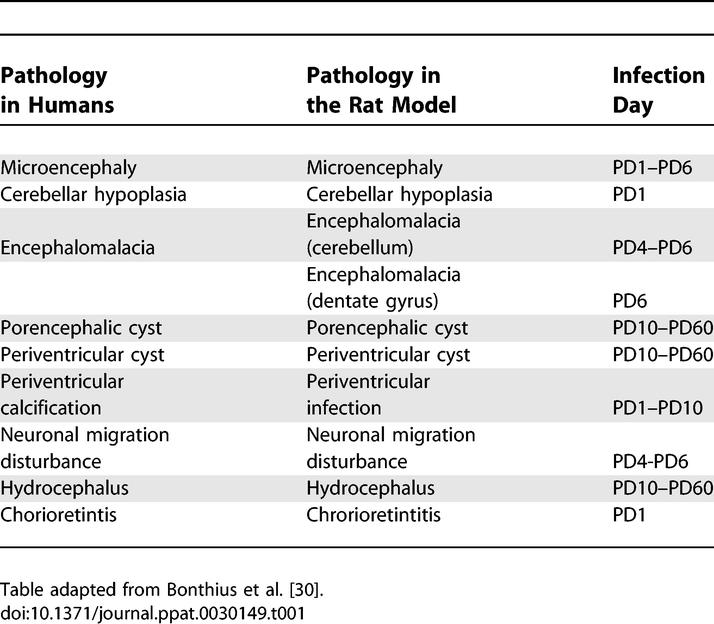
Pathology in Human Congenital LCMV Infection and in the Rat Model of the Disease

## Glial Cells Play a Critical Role in LCMV Infection of the Developing Brain

Glial cells play central roles in the entry, replication, and dispersion of LCMV in the developing rat brain [29]. Astrocytes and Bergmann glia are the initial parenchymal brain cells infected by LCMV and are the principal cell types in which LCMV replicates. Furthermore, it is via the sequential movement of virus from one astrocyte to its neighbor that LCMV spreads throughout the developing rat brain. Thus, glial cells are the portals of entry, the principal sites of replication, and the conduits through which LCMV disseminates through the brain parenchyma.

While glial cells are the major target of LCMV in the developing rat, this does not appear to be the case in developing mice, where neurons are the principal target [42]. Thus, the cellular targets of infection vary among host species. Whether glial cells are an important target of LCMV in the developing *human* brain is unknown.

## LCMV Preferentially Targets Neuroblasts

Following infection of glial cells, LCMV infects neurons. However, not all brain regions in the rat are vulnerable to neuronal infection. On the contrary, LCMV infects neurons only in four specific brain regions. These four regions are the cerebellum, olfactory bulb, dentate gyrus, and periventricular region [29]. Neurons elsewhere are spared.

Why LCMV infection of neurons is restricted to these four regions is unknown. One possibility is that neurons in these vulnerable regions selectively express a host cell receptor for the virus. Alpha-dystroglycan is a receptor for at least some strains of LCMV [43–45]. Whether alpha-dystroglycan is a receptor for LCMV within the brain is unknown. Alpha-dystroglycan is developmentally regulated in the rat brain [46], which, if it is the relevant receptor, may explain why the cellular targets of infection change with developmental age [30].

**Figure 5:**
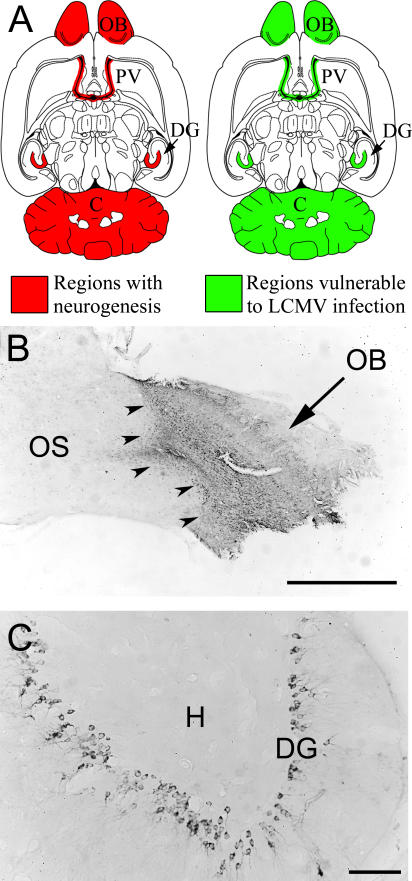
Within the Developing Brain, LCMV Induces a Specific Pattern of Infection, in which Brain Regions Containing Mitotically Active Neuronal Precursors Are Selectively Infected (A) During the early postnatal period in the rat, neurogenesis continues in four brain regions. These regions include the olfactory bulb (OB), periventricular region (PV), dentate gyrus (DG), and cerebellum (C). These four regions in which neurogenesis occurs are precisely the same four regions in which neurons are vulnerable to LCMV infection. (B) Micrograph illustrating the selective infection of the olfactory bulb by LCMV. This is a 50-μm-thick section through the anterior forebrain of a 30-d-old rat inoculated with LCMV on PD4. The section has been immunohistochemically stained for LCMV antigens. Note that the olfactory bulb (OB) is selectively infected. The immediately adjacent olfactory stalk (OS) is free of infection. The arrowheads demarcate the line between the olfactory bulb (right of arrowheads) and olfactory stalk (left of arrowheads) and demonstrate the highly selectivity pattern of LCMV infection. (C) Micrograph illustrating the selective infection of the dentate gyrus. This section was obtained from the same LCMV-infected animal shown in (B) and is immunohistochemically stained for LCMV antigen. Note that the neurons of the dentate gyrus (DG) are selectively infected, while the neurons of the immediately adjacent hilus (H) are entirely spared. Magnification bars represent 1 mm in (B) and 100 um in (C).

A second possibility is that the restricted pattern of neuronal infection reflects not the restricted ability of LCMV to *enter* cells, but to *replicate* within them. An important trait common to all four of the vulnerable brain regions in the neonatal rat is possession of at least one population of mitotically active neuronal precursors (Figure 5). Unlike all other brain regions in the neonatal rat, in which neuronal populations are uniformly post-mitotic, these four regions all possess neuroblasts that are generating new neurons [47–53]. Thus, LCMV infects neurons only in brain regions in which neurons are mitotically active. The converse is also true: all brain regions with mitotically active neuroblasts are infectable with LCMV [30]. This suggests that the metabolic machinery that accompanies neurogenesis promotes LCMV propagation.

The cerebral cortex is the exception that proves this rule. As described above, neurons of the cerebral cortex are infectable on PD1 and not thereafter. However, on PD1, cerebral cortical neurons are all post-mitotic [54]. Why, then, would they be infectable? Close inspection of the infection pattern within the cerebral cortex reveals that the cortical neurons are not uniformly infectable. Rather, a much greater proportion of neurons are infectable in the superficial cortical layers than in the deep cortical layers (Figure 4). During histogenesis, the cerebral cortex develops in an “inside out” sequence, so that neurons of the superficial cortical layers were generated *after* those of the deep cortical layers [55,56]. Thus, the neurons of the superficial cortical layers underwent mitosis more recently than those of the deeper layers and likely contain more of the metabolic machinery driving mitosis than do the deeper layers. Therefore, the “outside in” gradient of cortical LCMV infection reflects the “inside out” pattern of cortical neurogenesis and is exactly the pattern expected if LCMV neuronal infection depends upon neuronal mitotic machinery.

Another possibility is that LCMV replication depends not on neuronal mitotic activity per se but on an immature stage of neuronal differentiation. The brain regions that contain mitotically active neuroblasts also contain neurons at early stages of differentiation. Thus, multiplication of LCMV within neurons may depend upon cellular genes whose expression is restricted to the early stages of neuronal differentiation.

## LCMV Simultaneously Infects Multiple Brain Regions and Induces Different Forms of Pathology in Each Region

Following infection of neurons, LCMV induces neuropathological changes. However, the nature and time course of the neuropathology differ substantially among brain regions [29,30]. For example, when rat pups are inoculated with LCMV on PD4, the virus infects neurons of the cerebellum, olfactory bulb, dentate gyrus, and periventricular region and induces neuropathology unique to each region. In the cerebellum, LCMV induces an acute immune-mediated destruction of the dorsal lobules (Figure 2). This destructive process is driven by CD8+ lymphocytes, which infiltrate the cerebellum in large numbers and destroy the dorsal lobules. In the cerebellar ventral lobules, LCMV induces a neuronal migration disturbance, which causes cerebellar granule cells to be permanently ectopically located within the molecular layer (Figure 3).

In the olfactory bulb, LCMV infection at this same age leads to an acute hypoplasia of the olfactory bulb, due to a reduced production of granule cells. This hypoplasia is temporary, and the olfactory bulb can rebound to a normal size by adulthood [29].

In the hippocampal formation, a completely different pattern of neuropathology is observed. Here, the dentate gyrus initially appears histologically normal, despite a heavy viral burden within the dentate granule cells. However, several months post-inoculation, the previously infected granule cells begin to die. By 4 mo post-inoculation, there has been a profound loss of granule cells selectively from the dentate gyrus [29,57]. Thus, within the hippocampal formation, LCMV induces a delayed- onset selective mortality of dentate granule cells.

In the periventricular region, still a different pattern is observed. The periventricular region has neither an acute destructive process (like the dorsal cerebellum), nor an acute migration defect (like the ventral cerebellum), nor an acute hypoplasia (like the olfactory bulb), nor a delayed-onset drop-out of neurons (like the hippocampus). The periventricular region never shows any pathologic changes, despite infection of its neurons.

Why four brain regions simultaneously infected with a single viral species have such different pathologic responses is unknown. Part of the difference must be due to regionally different interactions with the immune system. Perhaps, in response to LCMV, the different brain regions produce different patterns of chemokines and cytokines, leading to regional differences in lymphocytic infiltration and different degrees of immune-mediated tissue injury. The regional differences in pathology may be due to differences in the host innate response to LCMV among brain regions. LCMV infection can trigger a robust innate immune response, including interferon expression and natural killer cell activation [58,59]. The innate immune response can differ in different parts of the brain. For example, microglia, which respond to initial insults to the central nervous system (CNS), are present in different densities throughout the CNS, which may result in region-specific pathological changes [60]. Thus, regional differences in the innate immune response may lead to different patterns and intensity of neuroinflammation, ultimately leading to different patterns of neuronal loss and other forms of tissue injury.

## LCMV-Induced Pathology of the Developing Brain Is Both Immune- and Virus-Mediated

The pathologic changes induced by LCMV infection of the developing brain are due to both the immune response and to the virus itself. Following inoculation of rats on PD4, LCMV induces destruction of the dorsal cerebellar lobules and hypoplasia of the olfactory bulbs [29]. The destructive process in the cerebellum is immune-mediated. Evidence for this lies in the facts that 1) the spatio-temporal pattern of tissue destruction corresponds perfectly with the spatio-temporal infiltration of lymphocytes [29], 2) suppression of the immune response with anti-lymphoid serum blocks the destructive process [38], and 3) the destructive process does not occur in congenitally athymic (nude) rats, which lack T lymphocytes [61].

In contrast, the olfactory bulb hypoplasia appears to be virus-mediated, as evidenced by the facts that the hypoplasia occurs in the absence of a lymphocytic infiltration following infection on PD4 [29] and that an identical hypoplasia occurs following infection on PD1 [30], a time when the animal is tolerant to the virus and does not mount an immune response to it.

Whether the pathology in any particular brain region is immune-mediated or virus-mediated, however, depends on the age of the host at the time of infection [30]. In the cerebellum, LCMV induces immune-mediated destruction if the infection occurs on PD4, but virus-mediated hypoplasia if the infection occurs on PD1. In the olfactory bulb, LCMV induces virus-mediated hypoplasia if the infection occurs on PD4, but immune-mediated destruction if the infection occurs on PD6 or later. Thus, different brain regions simultaneously infected with LCMV can undergo different forms of pathology with different underlying mechanisms, and the type of pathology in any particular brain region can shift from virus-mediated to immune-mediated, depending on host age at the time of infection [30].

## Speculations and Future Directions for Research

Despite recent advances, much remains unknown regarding the biology of LCMV infection of the developing brain. Why there is so much variability in the pathology and outcome among cases of human congenital LCMV infections is one key question [11]. As discussed above, differences in gestational timing of infection probably account for much of the variability. But other factors may contribute, as well. Children with congenital LCMV infection may have variable outcomes because they were infected with different viral strains. Different strains of LCMV have different avidities for the receptor alpha-dystroglycan [45]. In the adult mouse spleen, these different viral strains have distinct infection kinetics and tissue tropisms, and they cause distinct diseases [62]. The same may be true in the developing brain.

Another possible explanation for variability in outcome is differences in host genetics. LCMV can alter gene expression within neurons and thereby induce neuronal dysfunction [63,64]. Genetic differences among individuals may underlie different host gene–LCMV interactions and lead to different patterns of neurological dysfunction [65,66].

How LCMV induces a neuronal migration defect is another important question with clear clinical implications. Within the rat's developing cerebellum, LCMV disrupts the migration of granule cells from the external granule cell layer to the internal granule cell layer and leaves many of the cells permanently ectopic within the molecular layer [29]. Cerebellar granule cells are a major target of LCMV [29–32]. Thus, the migration defect may be due to a direct effect of the virus on the migrating cells. However, LCMV also heavily targets Bergmann glia [29], which constitute the scaffolding along which granule cells migrate [67,68]. Thus, the migration defect may be due to a virus-induced corruption of Bergmann glia structure or function. A third possibility is that the migration defect is due to an alteration in the chemical environment of the cerebellum. During migration, cerebellar granule cells respond to gradients of chemicals, including chemokines, to direct their migratory movements [69,70]. LCMV infection and its accompanying inflammatory response likely alter the chemical environment, including chemokine concentrations, which may misdirect the forward progress of migrating neurons. Recently, in vitro systems have been developed that allow systematic study of the role of Bergmann glia, cell surface molecules, and diffusible substances in cerebellar granule cell migration [71]. Application of these systems may elucidate the relative roles of cytokines, chemokines, altered Bergman glia, and altered granule cell function in the neuronal migration disturbance induced by LCMV.

LCMV infection of the developing rat leads to substantial regional differences in pathology across the brain. Similarly, humans with congenital LCMV infection can have different forms and severity of pathology in different brain regions [11]. The differences in pathology are not due to regional differences in viral titer, since titers are often similar among regions with substantially different pathologies [29,30]. One possibility, as discussed above, is regional differences in cytokine and chemokine production leading to regional differences in lymphocytic infiltration and tissue destruction. Another possibility is regional differences in glial cell biology. Astrocytes perform multiple functions vital to the health of their neuronal neighbors and are a principal target of LCMV [29]. Furthermore, astrocytes are not homogeneous across the brain. Substantial regional differences exist in glial cell physiology [72–75]. Thus, regional differences in LCMV-induced pathology may be due to regional differences in astrocytic responses to the virus.

One of the most fascinating aspects of LCMV infection occurs in the developing hippocampal formation. Following inoculation on PD4, the dentate gyrus is selectively and heavily infected [29,57]. Despite a heavy viral load, the dentate gyrus suffers no acute injury, and a full complement of histologically healthy-appearing dentate granule cells is generated. However, several months post-inoculation, a selective drop-out of dentate granule cells begins [31]. This delayed-onset cellular mortality progresses over months and eventually results in a severe depletion of dentate granule cells [57] and a corresponding decrease in the seizure threshold [76]. Importantly, the virus has been cleared from the animal *before* much of the neuronal loss occurs [29,57]. Thus, LCMV infection in the neonatal rat brain initiates a pernicious and progressive pathological phenomenon that continues even after the virus has been cleared. The mechanism underlying this delayed-onset cell loss is unknown, but may be due to excitotoxicity. Dentate granule cells previously infected with LCMV are electrophysiologically abnormal and hyperexcitable [57]. Furthermore, GABAergic (inhibitory) interneurons of the dentate gyrus are infected with LCMV before the loss of dentate granule cells begins [77]. Thus, the delayed-onset loss of dentate granule cells may be due to viral-induced disruption of inhibitory circuits that persists even after the virus is gone.

Many prominent childhood- and adult-onset neurological and psychiatric diseases are hypothesized to be due to viral infections that occurred at a much younger age. Such diseases include Alzheimer disease, Parkinson disease, multiple sclerosis, temporal lobe epilepsy, schizophrenia, bipolar disorder, and autism [78–83]. The fact that LCMV infection of the neonatal rat brain leads to delayed-onset neuronal loss after the virus has been cleared establishes the neonatal rat model of LCMV as a potentially valuable model system for the study of many important neurological diseases whose etiology has remained elusive.

The pathogenesis of congenital LCMV infection in humans is a mystery. However, because the neonatal rat model so reliably reproduces the pathology observed in humans, use of the animal model holds great promise for understanding the biology and pathogenesis of this emerging infectious disease. Lessons learned from the LCMV-infected rat may also shed light on the pathogenesis of other congenital infections.

## Abbreviations

LCMV: lymphocytic choriomeningitis virus
PD: postnatal day
